# Electroporation (EP)-related technical errors experienced during an HIV vaccine clinical trial conducted in Rwanda and Uganda: lessons learned

**DOI:** 10.1186/1742-4690-9-S2-P113

**Published:** 2012-09-13

**Authors:** R Bayingana, A Nanvubya, E Karita, J Nyombayire, R Ingabire, K Chinyenze, J Lehrman, C Schimidt, D Hannaman, S Allen, P Fast

**Affiliations:** 1 Project San Francisco/International AIDS Vaccine Initiative, Kigali, Rwanda; 2 Uganda Virus Research Institute, Entebbe, Uganda; 3 International AIDS Vaccine Initiative, Nairobi, Kenya; 4 ICHOR Medical Systems Inc, USA; 5 Emory University, Atlanta, GA, USA

## Background

Intracellular DNA vaccine delivery is essential for antigen expression and induction of immune response. Unfortunately, conventional intramuscular injection provides low efficiency DNA uptake and suboptimal immunogenicity. Electroporation-based DNA vaccination can enhance potency by 100-1000 fold. In this trial we used the TriGrid Delivery System by Ichor Medical Systems. We describe the electroporation technical errors that occurred during administration of an HIV pDNA vaccine in a clinical trial conducted at sites in Rwanda and Uganda and how those errors were resolved.

## Methods

The site physicians were trained and qualified to use the EP procedure during site initiation visits and further practiced mock EPs later. We examined the technical errors that occurred by reviewing volunteer files between 19th December 2011 and 27th March 2012.

Each administration of the HIV pDNA intramuscularly by electroporation required two injections (one in each deltoid muscle) at each vaccination time point. Should an error occur during EP procedure, the respective error codes are shown on the monitor. Once the procedure is complete, ‘procedure complete’ is indicated on the monitor.

## Results

Six errors out of 90 eletroporations occurred. Four occurred because of improper insertion of the electrodes or injection needle. These errors were resolved by repeating the procedure. Two occurred because of improper insertion of the cartridge into the applicator and on both occasions the errors were captured before switching on the enable button on the pulse stimulator. These were resolved by properly re-inserting the cartridge into the applicator. Most of the other potential errors were caught before the procedure started by the second person observing the set up.

## Conclusion

We conclude that errors related to EP procedure can be minimized or avoided when there is appropriate quality control immediately prior to vaccination. We advise to have two trained and qualified staff present during the procedure for quality control purposes.

